# Reversible phase transformations between Pb nanocrystals and a viscous liquid-like phase

**DOI:** 10.1126/sciadv.adn6426

**Published:** 2024-06-19

**Authors:** Wenjing Zheng, Jun Kang, Kaiyang Niu, Colin Ophus, Emory M. Chan, Peter Ercius, Lin-Wang Wang, Junqiao Wu, Haimei Zheng

**Affiliations:** ^1^ School of Energy and Power Engineering, North University of China, Taiyuan 030051, China.; ^2^ Beijing Computational Science Research Center, Beijing 100193, China.; ^3^ Department of Physics, Beijing Normal University, Beijing 100875, China.; ^4^ Materials Sciences Division, Lawrence Berkeley National Laboratory, Berkeley, CA 94720, USA.; ^5^ National Center for Electron Microscopy, Molecular Foundry, Lawrence Berkeley National Laboratory, Berkeley, CA 94720, USA.; ^6^ The Molecular Foundry, Lawrence Berkeley National Laboratory, Berkeley, CA 94720, USA.; ^7^ Institute of Semiconductors, University of Chinese Academy of Sciences, Beijing 100083, China.; ^8^ Department of Materials Science and Engineering, University of California, Berkeley, Berkeley, CA 94720, USA.

## Abstract

Phase transformations have been a prominent topic of study for both fundamental and applied science. Solid-liquid reaction–induced phase transformations can be hard to characterize, and the transformation mechanisms are often not fully understood. Here, we report reversible phase transformations between a metal (Pb) nanocrystal and a viscous liquid-like phase unveiled by in situ liquid cell transmission electron microscopy. The reversible phase transformations are obtained by modulating the electron current density (between 1000 and 3000 electrons Å^−2^ s^−1^). The metal-organic viscous liquid-like phase exhibits short-range ordering with a preferred Pb-Pb distance of 0.5 nm. Assisted by density functional theory and molecular dynamics calculations, we show that the viscous liquid-like phase results from the reactions of Pb with the CH_3_O fragments from the triethylene glycol solution under electron beam irradiation. Such reversible phase transformations may find broad implementations.

## Teaser

Liquid cell TEM study unveils reversible phase transformations between Pb metal nanocrystals and a viscous liquid-like phase.

## INTRODUCTION

Phase transformations of materials are of key importance across the field of science and technology (*1*, *2*). Materials undergoing phase transformations are often accompanied with property changes; thus, they may have various implementations ranging from sensing (*3*, *4*) to energy storage (*5*, *6*), thermal energy conversion (*7*–*9*), drug delivery (*10*, *11*), and biological applications (*12*, *13*).

Phase transformations are often associated with overcoming a distinct energy barrier. There have been extensive studies on phase transformations to resolve the structural changes of materials under various stimuli, such as temperature changes (*14*, *15*), applied electric fields (*16*–*18*), light (*19*, *20*), electron beams (*21*), or others (*22*, *23*). When the energy barrier is sufficiently small that it is comparable with the energy for thermal fluctuations (*21*), spontaneous phase transformations fluctuating back and forth between two phases may occur.

Many solid-solid phase transformations are achieved through local atomic re-arrangements within the solids and are reversible (*24*, *25*). However, for structural transformations through solid-liquid chemical reactions, it is often irreversible because of the structural and chemical heterogeneity at solid-liquid interfaces (*26*, *27*), or mass loss due to reactants diffusing away. For example, in applications of Si nanostructures in lithium-ion batteries, the Si anode materials react with the lithium ions in the electrolyte to form SiLi*_x_* accompanied by large volume changes (*28*, *29*). During the charge/discharge processes, the nanostructure shatters and the mass loss makes the original Si nanostructure unrecoverable, leading to degradation of the devices (*30*, *31*). Additionally, structures that result from solid-liquid reactions can be complex and challenging to resolve. Exploring reversible solid-liquid phase transformations and developing a fundamental understanding of the transformation mechanisms are crucial for future technological applications. However, it is a great challenge to directly observe reversible solid-liquid phase transformation dynamics due to experimental limitations.

Here, we report reversible phase transformations between Pb metal nanocrystals and a unique viscous liquid-like phase discovered through in situ liquid cell transmission electron microscopy (TEM). Liquid cell TEM is a powerful tool to study nanoscale structural transformations of materials (*32*). Compared to ensemble x-ray diffraction and Raman spectroscopy, liquid cell TEM has the advantage of investigating individual nanoparticles with high spatial resolution (*33*–*35*). Here, we use liquid cell TEM to directly monitor nanocrystal transformations in a custom liquid cell (*33*), in which the electron beam is used to initiate and manipulate nanocrystal transformations. Reactions of Pb (or Bi) nanocrystals with a solution of triethylene glycol (TEG) under the electron beam irradiation generates a viscous liquid-like phase. Structural characterizations of the viscous liquid-like phase are achieved using electron energy loss spectroscopy (EELS) and high-resolution TEM (HRTEM). Moreover, we further conduct density functional theory (DFT) and molecular dynamics (MD) simulations to understand the mechanisms underlying the reversible transformations between metal nanocrystals and the viscous liquid-like phase.

## RESULTS

### As-synthesized Pb nanocrystals

**Fig. 1.**
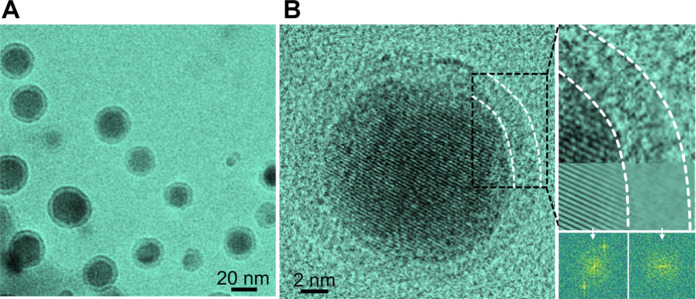
Synthesis of Pb nanocrystals in the liquid cell TEM. (**A**) Low-magnification TEM image of core-shell nanoparticles (movie S1). (**B**) HRTEM images of a Pb nanocrystal, and the corresponding FFT patterns and inversed FFT images.

We prepare Pb nanocrystals by in situ synthesis in a liquid cell under TEM (details in Materials and Methods). The precursor solution is obtained by mixing lead (II) acetylacetonate and TEG. TEG is a typical carrier for pharmaceutical applications and it is also a reducing agent for polyol synthesis of metal nanostructures (*36*, *37*). The precursor solution is loaded into a SiN*_x_* liquid cell (*33*) (fig. S1), and nanoparticle formation is initiated by electron beam irradiation. It involves the formation of dense liquid domains followed by nucleation and growth of crystalline nanoparticles (see details in fig. S2 and movie S1). Eventually, Pb nanocrystals wrapped with a 2-nm-thick amorphous shell are obtained (Fig. 1A). The sizes of the core-shell nanoparticles range from 15 to 30 nm. The nucleation and growth processes share some similarities with the two-step nucleation of gold clusters (*38*), and the dense solution is likely the precursor solution with higher concentration of metal ions (*39*).

The core-shell nanoparticles are further characterized using HRTEM and energy-dispersive x-ray spectrometry (EDS), as shown in Fig. 1B and fig. S3 (A and B). The HRTEM image and the corresponding fast Fourier transform (FFT) patterns show that the core is crystalline Pb and the shell is an amorphous phase (Fig. 1B). Because of the rotations of nanocrystals, the core-shell structure with the intact encapsulation of Pb nanocrystal core by a soft liquid-like shell can be confirmed (movie S2 and fig. S4). EDS spectrum of the core-shell nanoparticles indicates that they are Pb. It may be surprising that oxygen content is negligible from the EDS spectrum, since the amorphous liquid-like shell is considered to be composed of Pb with solution molecules or ions that may contain oxygen (*40*). However, due to the fact that the shells are very thin and the nanoparticles are electron beam sensitive, further characterization is needed to resolve the shell composition and structure of the as-synthesized nanoparticles.

### Reversible phase transformations between Pb nanocrystals and a viscous liquid-like amorphous phase

When the electron beam is converged on an as-synthesized Pb nanocrystal in the liquid cell, the Pb nanocrystal expands and transforms into an amorphous phase. Subsequently, as the electron beam is spread, the nanocrystal immediately recovers its original crystallinity, size, and shape (movie S3 and fig. S5). Considering this remarkable transformation behavior of Pb nanocrystals upon electron beam current density changes, we conduct systematic studies to examine the transformations between a crystalline Pb solid and a viscous liquid-like amorphous phase.

**Fig. 2.**
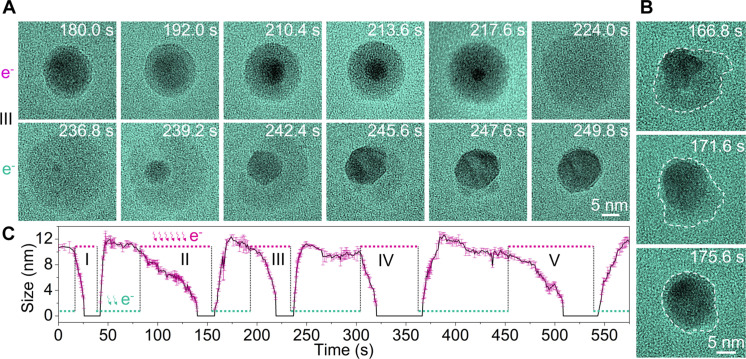
Reversible phase transformations of nanocrystals between a crystalline Pb solid and a viscous liquid-like amorphous phase. (**A**) TEM image series (with false color) showing the transformation from a crystalline Pb solid state to a viscous liquid-like amorphous state under a strong beam (3000 electrons Å^−2^ s^−1^), and then it transforms back to the solid state under a weak beam (1000 electrons Å^−2^ s^−1^). (**B**) Specially selected sequential TEM images with highlighted contours of the amorphous shell. The flexible changes of shell configuration indicate that the amorphous shell is liquid-like. Most of the time, the shell is spherical likely due to surface tension. (**C**) Five cycles of reversible phase transformations as reflected in the plot of Pb nanocrystal core size evolution with time corresponding to the repeated switching between the strong beam and the weak beam (movie S3).

**Fig. 3.**
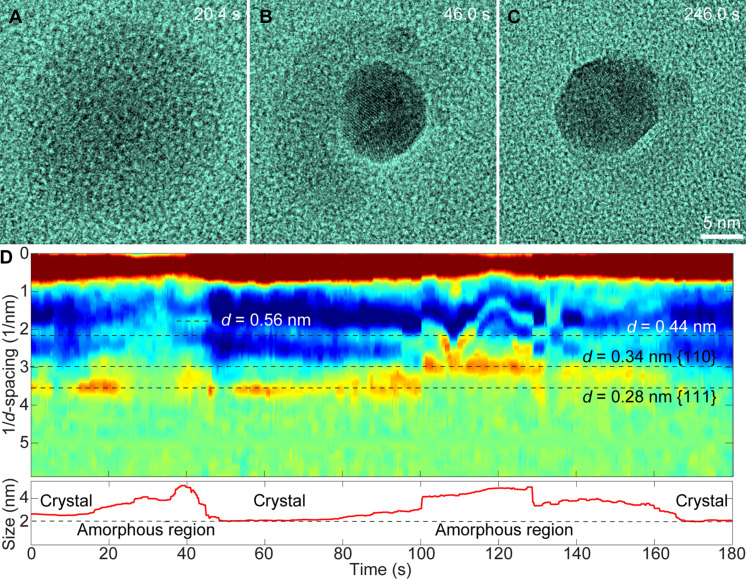
Structure determination of Pb nanoparticles with the crystalline solid and viscous liquid-like phase. (**A**) HRTEM image of the viscous liquid-like state of the Pb nanoparticle (20.4 s). (**B** and **C**) Pb nanocrystal with the viscous liquid-like phase around from viscous liquid-like amorphous phase (46.0 and 246.0 s). (**D**) Determination of the lattice spacing of the Pb nanoparticle over the reversible transformations. The viscous liquid-like phase (labeled as amorphous region) show substantial scattering intensities corresponding to *d*-spacing of 0.44 and 0.56 nm.

Under high-resolution imaging, we switch the electron beam current density between ~3000 electrons Å^−2^ s^−1^ (strong beam) and ~1000 electrons Å^−2^ s^−1^ (weak beam) by converging and spreading the beam repeatedly. As shown in Fig. 2A, the initial Pb crystalline nanocrystal with a thin amorphous shell transforms into an amorphous phase under the strong beam of 3000 electrons Å^−2^ s^−1^. The transformation proceeds by the crystalline Pb core shrinking and the amorphous shell growing. Eventually, the whole nanoparticle turns into an amorphous phase after 44 s (at 224.0 s). Subsequently, under the weak beam of 1000 electrons Å^−2^ s^−1^, a Pb nanoparticle nucleates from the amorphous phase and it grows by consuming the amorphous phase (see more details in fig. S6 and Fig. 3, B and C). After 13 s, it transforms to the original state of Pb nanocrystal with a thin amorphous shell (at 249.8 s). The Pb core maintains crystalline during phase transformations, as shown in the HRTEM images (Fig. 3, B and C, and will discuss further). The amorphous phase behaves like a viscous liquid, for example, the shell configuration changes frequently with soft curvature (Fig. 2B). However, most of the time, it is spherical likely due to surface tension. Many cycles of reversible phase transformations are achieved under repeatedly switching the beam between a strong beam and a weak beam. We plot the Pb core size (projected area) during phase transformations with time, which reflects cycles of the reversible phase transitions (Fig. 2C). The duration of each transformation varies, which likely results from differences in the beam conditions due to manual switching of electron beam intensity. TEM images of five cycles of phase transformations are shown in fig. S7. It shows the crystalline Pb nanocrystal after each cycle of phase transformation has a similar size.

The phase transformations from viscous liquid-like amorphous phase to crystalline Pb solid display commonly observed phenomena in nucleation and growth of nanocrystals. For example, single or multiple nucleation events, coalescence of nanoparticles to form polycrystalline structure, formation of a single crystal with distinct facets, and so on have been found (Fig. 3, A to C, and fig. S7). The crystal lattices of Pb nanocrystals during phase transformations in both directions can be clearly identified from the high-resolution images. We also carefully examine any possible structural ordering of the viscous liquid-like amorphous phase, as it follows.

For each high-resolution image in a movie, we calculate the scattering intensity from the nanoparticles in the Fourier transform of each image frame. Since the microscope transfer function (MTF) modulates the information transform, and various factors (e.g., sample motion, microscope defocuses, and astigmatism settings) modify the MTF, the impacts from MTF should be removed in calculating the scattering intensity.

The MTF can be easily measured in Fourier space when an amorphous sample is present in the field of view, by fitting the Thon ring features (*41*, *42*). Here, we have used custom MATLAB code to fit the MTF of the Fourier transform amplitude of each image frame, as well as a smoothly varying background intensity modeled as a two-dimensional Gaussian. Then, the scattering intensity at each pixel is normalized by subtracting the MTF, and radially integrated to give the estimated intensity at each scattering vector. These values were plotted as a function of time in Fig. 3D (more details in fig. S8). The results show that both the amorphous and crystalline phases produce substantial amplitude contrast due to their higher average atomic number, leading to a local decrease in the image intensity. The calculated lattice d-spacing of 0.28 and 0.34 nm correspond to the {111} and {110} lattice planes of the crystalline lead (space group *Fm*$\bar{3}$*m*, 225; *a* = 4.99 Å). It is also noted that there are substantial scattering intensities with d-spacing of 0.44 and 0.56 nm from the viscous liquid-like amorphous phase, which suggests structural ordering.

### Control experiments

**Fig. 4.**
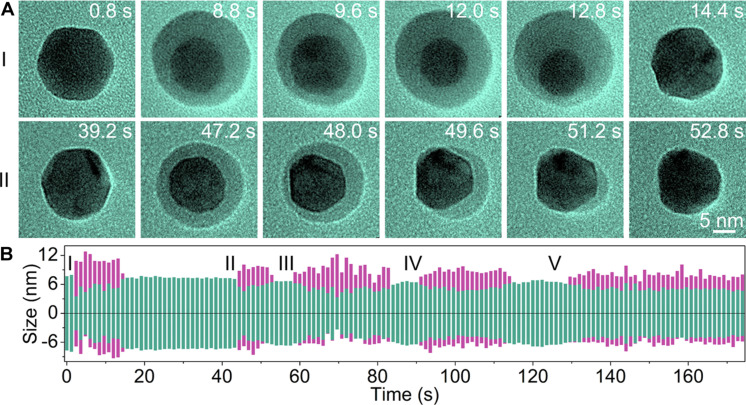
Reversible Pb nanocrystal transformations under random electron beam manipulations (movie S4). (**A**) TEM image series of a Pb nanocrystal that transforms rapidly under a random electron beam, where the viscous liquid-like phase forms or disappears instantaneously when the electron beam is converged or spread on the Pb nanocrystal. (**B**) Dimension evolution of the crystal phase (magenta) and the viscous liquid-like phase (green) under random electron beam manipulations.

Since the reversible phase transformations of a crystalline Pb solid and a viscous liquid-like amorphous phase are driven by electron beam irradiation, we further conduct a series of experiments using the electron beam to control the phase transformations. We achieve partial transformations of the Pb nanocrystals by first increasing the electron beam intensity and then quickly reducing it to ~1000 electrons Å^−2^ s^−1^ before the Pb nanocrystal is completely transformed into the amorphous phase. Core-shell nanocrystals with Pb nanocrystal core and viscous liquid-like amorphous shell are obtained during the transformations, while Pb nanocrystals are found at the beginning and end (Fig. 4A; also see fig. S9 and movie S4). Figure 4B shows the sizes of the Pb core (magenta) and viscous liquid-like amorphous shell (green) with time while the beam current density changes frequently. Details of the measurements for Fig. 4B are shown in fig. S9C.

We also vary the beam current density between 3500 and 2000 electrons Å^−2^ s^−1^, and notable different transformation dynamics are observed in the transformation from the viscous liquid-like amorphous to crystalline solid (movies S5 and S6 and figs. S10 and S11). There are many nuclei, and the growth rate is much slower than those under 1000 electrons Å^−2^ s^−1^. It takes much longer time to complete the transformation to Pb crystalline solid, e.g., 100 s under 2000 electrons Å^−2^ s^−1^ (~50 s under 1000 electrons Å^−2^ s^−1^).

Additionally, under the same beam intensity, smaller Pb nanoparticles transform faster than larger ones (figs. S12 and S13 and movie S7). All phase transformations occur in the liquid solution with TEG (fig. S14 and movie S8).

Similar reversible phase transformations between a crystalline solid and an amorphous phase are found in Bi nanoparticles (figs. S15 and S16).

### Theoretical calculation of the Pb-TEG system

**Fig. 5.**
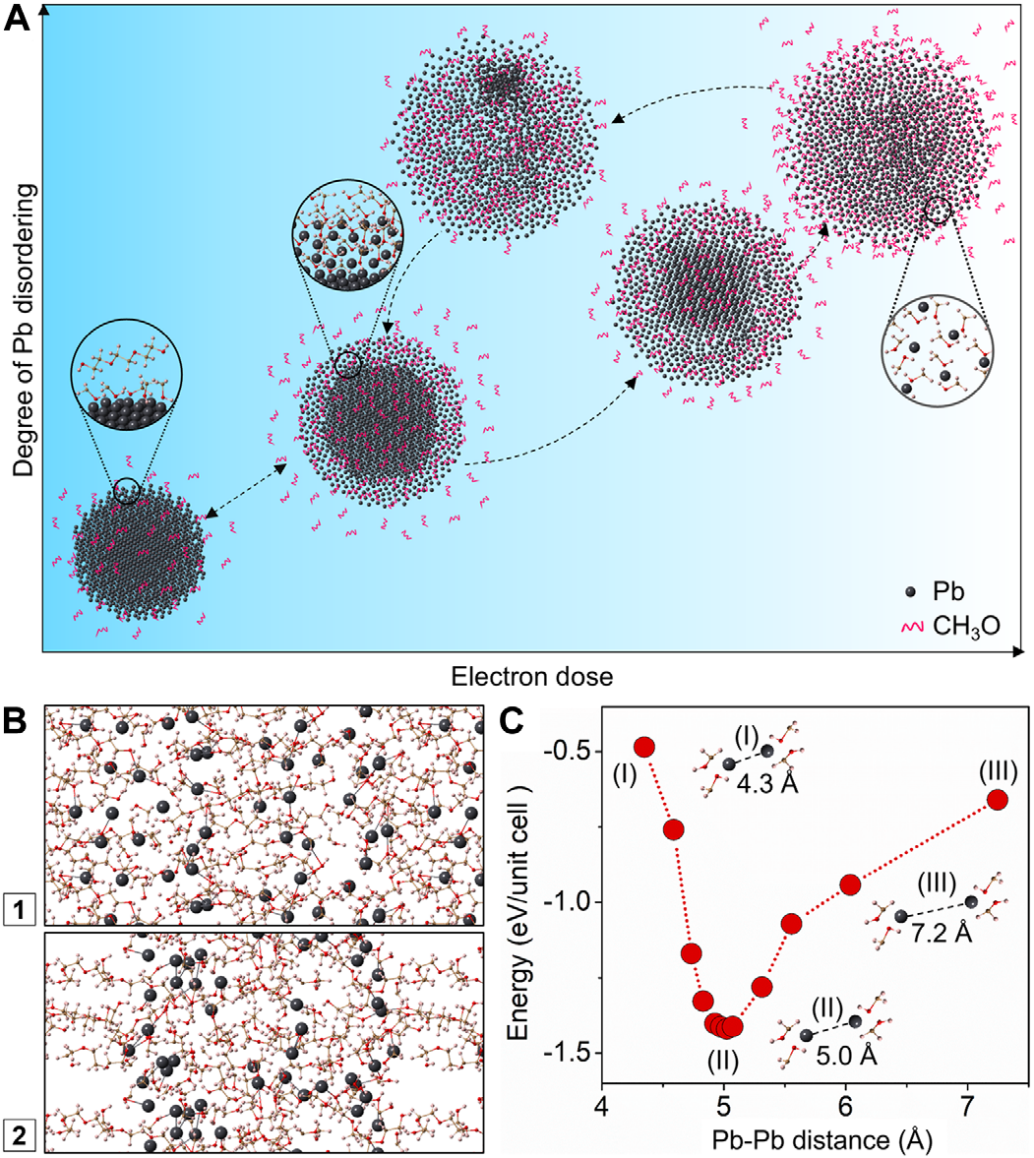
A schematic illustration of phase transformations between a Pb nanocrystal and viscous liquid-like structure, and theoretical calculations. (**A**) Illustration of the degree of Pb disordering at different state of phase transformations. (**B**) MD simulation of Pb(CH_3_O)_2_ clusters in TEG matrix. Scenario 1 displays the initial structure where Pb(CH_3_O)_2_ clusters are randomly distributed in the TEG matrix. Scenario 2 shows the structure after 6.5-ps MD relaxation where Pb(CH_3_O)_2_ tends to aggregate, instead of mixing with TEG. (**C**) Energy states of Pb(CH_3_O)_2_ as a function of Pb-Pb distance. The energy of an isolated Pb(CH_3_O)_2_ is set to zero.

As demonstrated in the above in situ experiments, Pb nanocrystals transform to viscous liquid-like structure under the strong electron beam irradiation. The viscous liquid-like phase transforms back to Pb nanocrystals when the electron beam intensity is reduced. This suggests that Pb nanocrystals react with molecular species in the solution and form viscous liquid-like structure with the external energy input (strong beam). The viscous liquid-like phase is metastable, transforming back to the more stable phases of Pb solid and organic species as the extra energy input is removed (weak beam). Figure 5A illustrates the transformation pathways between Pb nanocrystal and viscous liquid-like phase. To further understand the reversible structural transformations, we perform DFT calculation and MD simulation.

TEG molecules can break down into small fragments under the strong electron beam irradiation (see more EELS characterization in figs. S17 and S18). The bond breaking is expected to occur at the C─C bonds (*43*, *44*). Thus, majority of the small fragments will be CH_3_O. Other fragments can also be obtained, for example, C_2_H_4_O will be produced when all the C─C bonds in TEG are broken. We evaluate the possible reactions of these fragments with other species in the solution. For the CH_3_O fragments, they may involve in three possible reactions: (i) The CH_3_O fragments may bind with acac, leading to the formation of Pb quantum dots: Pb(acac)_2_ + 2CH_3_O → Pb + 2(CH_3_O)(acac). (ii) CH_3_O may react with the Pb quantum dots, forming a Pb-CH_3_O mixture (in a 1:2 ratio): Pb + 2CH_3_O → Pb(CH_3_O)_2_. (iii) Recovery of Pb quantum dots: Pb(CH_3_O)_2_ → Pb + (CH_3_O)_2_. Our DFT calculation results show that the reaction energy is −2.00 eV/Pb for (i), −1.54 eV/Pb for (ii), and −2.85 eV/Pb for (iii), respectively. Small modifications of the calculated reaction energy are obtained when the solvent effects are considered in the DFT calculation (Supplementary Materials). It is clear that it is energetically most favorable for CH_3_O to bind with each other to form TEG [here we use (CH_3_O)_2_ to represent the recovery of C─C bonds], and the Pb atoms bind together to form Pb quantum dots.

Considering the C_2_H_4_O fragments, we also did calculations to evaluate the following reactions: (iv) Pb(acac)_2_ + C_2_H_4_O → Pb + (acac)_2_C_2_H_4_O and (v) Pb + C_2_H_4_O → Pb(C_2_H_4_O). The calculated reaction energy is 1.41 eV/Pb for (iv) and 1.84 eV/Pb for (v). Since the energies are positive, these reactions are thermodynamically unfavorable. Therefore, we conclude that the CH_3_O fragments possibly participate in the formation of viscous liquid-like phase, while the C_2_H_4_O fragments should have negligible effects on the phase transformations. The formation of viscous liquid-like phase [Pb(CH_3_O)_2_] from reaction (ii) requires the additional energy input (e.g., strong beam) compared to other two reactions.

We further use MD simulation to evaluate the Pb(CH_3_O)_2_ phase. We establish a model by randomly putting 32 Pb(CH_3_O)_2_ molecules and 20 TEG molecules in a 17 Å × 17 Å × 40 Å box, and the temperature is set to 500 K. As shown in Fig. 5B, after 6.5 ps, segregation of Pb(CH_3_O)_2_ from the TEG is clearly seen. This suggests that Pb(CH_3_O)_2_ can form an immiscible phase as it does not dissolve in the TEG solvent.

Additionally, we build a cuboid unit cell that contains one Pb(CH_3_O)_2_ molecule. The lattice constant of the unit cell is then optimized, and the average Pb-Pb distance *d* is estimated by ∛*V*, where *V* is the volume of the unit cell. The relationship between the total energy of the unit cell and the average Pb-Pb distance can be calculated. As shown in Fig. 5C, the Pb-Pb distance of 5 Å has the minimum energy, implying that the structure of Pb(CH_3_O)_2_ phase is not totally random but with a preferred Pb-Pb distance of 5 Å.

## DISCUSSION

Our direct observation of nanocrystal transformations reveals reversible phase transformations between Pb nanocrystals and a unique viscous liquid-like phase, which has been assigned to a Pb(CH_3_O)_2_ structure. As shown in Fig. 5A, a core (Pb)–shell [Pb(CH_3_O)_2_] structure can be found along the pathways of transformations. The viscous liquid-like phase shows short-range ordering with the Pb-Pb distance of ~0.5 nm.

The viscous liquid-like structure [Pb(CH_3_O)_2_] results from Pb reacting with CH_3_O fragments from TEG molecules under electron beam irradiation. The Pb↔Pb(CH_3_O)_2_ transformations are highly sensitive to the electron beam intensity variations. On the basis of our DFT calculation, the reactions involving CH_3_O fragments are all energetically favorable [negative values of reaction energy for the (i) to (iii) reactions] with small differences in their energy level (fig. S19). Thus, with the extra energy input (e.g., under a strong beam), reaction (ii) can occur, leading to the formation of Pb(CH_3_O)_2_ structure. When the energy is released (e.g., under a weak beam), reaction (iii) is preferred, which results in the recovery of Pb quantum dots and TEG.

Last, we have found similar structures in other metal nanoparticle systems (e.g., Bi) under electron beam irradiation or in electrochemical reactions, suggesting that our study of reversible transformations may have broad implementations. Insights gained from this study may assist future applications of metal nanoparticles in sensing, drug delivery, or others.

## MATERIALS AND METHODS

### Materials

All chemicals including Pb(acetylacetonate)_2_ (99%, Aldrich), TEG (99%, Aldrich), lead nitrate (Pb(NO_3_)_2_, Aldrich), sodium stearate (NaSt, Aldrich), oxtanol (OcOH, Aldrich), and toluene (Aldrich) were used as received.

### Liquid cell fabrication and growth solution loading for TEM

Liquid cells are fabricated by following a similar process as described in a previous publication (*32*). We use ultrathin silicon wafers (100 μm, 4 inches, p-doped) purchased from Virginia Semiconductor (Fredericksburg, VA) and deposited low-stress silicon nitride membranes with a thickness of only 13 nm on the silicon wafers. Here, the use of ultrathin silicon nitride membranes has effectively improved the spatial resolution of the liquid cell to the subnanometer range. The subsequent fabrication processes include lithographic patterning, wet KOH etching of silicon, and liquid cell bonding using an indium thin film spacer. The indium thin film is deposited by sputtering, and it acts as a spacer as well as the sealing material for the liquid cell. 100 nm spacing is used for the current experiments, although the different thicknesses can be achieved. All the fabrication processes are conducted at the Nanofabrication Lab of the University of California at Berkeley. The liquid loading is facilitated by a syringe and Teflon nanotube (purchased from Cole-Parmer, Vernon Hills, IL) to control the size of the liquid droplet. A droplet of 30 pl is directed into the liquid reservoir without contaminating the electron transmission window.

After the liquid is loaded into the liquid cell, we cover the cell using a single slot copper TEM grid (TEM grids are purchased from Ted Pella Inc.) and seal the whole liquid cell using epoxy. Properly sealing the liquid cell can assist in maintaining the liquid inside the liquid cell for an extended period of time, which is critical for enabling the reversible nanocrystal transformation.

### Initiation of lead nanoparticle growth

The growth of lead nanocrystals in a liquid cell is initiated by the electron beam illumination of the growth solution. The reduction of Pb(acetylacetonate)_2_ precursor (Pb^2+^) to metal (Pb^0^) can be from TEG fragment-assisted metal ion reduction.

### TEM imaging and image processing

All movies were recorded under JEOL 2100 TEM with a high-resolution pole piece (Cs = 1 mm) and a LaB_6_ filament using a Gatan Orius charge-coupled device (CCD) camera. The movie was recorded at a rate of 5 frames/s by the open-sourced software VirtualDub embedded in the DigitalMicrograph software. The as-recorded movie was compressed to reduce the file size. All movies play faster than in real time and are compressed to larger pixel sizes to reduce the file sizes.

Nanocrystals in the sequential images in Figs. 1 to 4 are highlighted in green by a false coloring process using Photoshop software. All original images can be retrieved (see movies S1 to S8).

### Beam dose estimation

$$\text{Beam dose rate}=\frac{\text{Total pixel counts}}{\text{Conversion efficiency}\times \text{Area}\times \text{Exposure time}}$$

The electron beam (200 kV; beam current density of about 500 electrons Å^−2^ s^−1^) passes through the silicon nitride window (3 μm × 50 μm) and induces the growth of Bi nanoparticles in the liquid layer.

### DFT calculations

DFT calculations were performed using the Vienna AB Initio Simulation Package (VASP) (*45*). The core-valence interaction was described by the projector-augmented wave (PAW) method (*46*), and the generalized gradient approximation of Perdew-Burke-Ernzerhof (GGA-PBE) (*47*) was used. The wave functions were expanded in a plane-wave basis set with a 400-eV cutoff. Structures were relaxed until the force acting on each atom was less than 0.01 eV/Å. Van der Waals interactions were included by using the empirical correction scheme of Grimme (*48*). To verify the mechanism of the reactions, the calculations are performed using isolated molecules in a vacuum. However, in the experiment, the reactions occur under a solvation environment. To further verify the calculations, we also calculated the reaction energy using an implicit solvation model that describes the effect of electrostatics, cavitation, and dispersion on the interaction between a solute and solvent. The dielectric constant of 23.0 for TEG solvent is used.

### MD simulation

To study the segregation of Pb(CH_3_O)_2_ and TEG, 32 Pb(CH_3_O)_2_ molecules were uniformly dispersed in 20 TEG molecules. The box size was 17 Ang × 17 Ang × 40 Ang. The temperature is set to 500 K.
