# Supplementary material for: Reversible phase transformations between Pb nanocrystals and a viscous liquid-like phase
Source: Sci Adv. 2024 Jun 19;10(25):eadn6426. doi: 10.1126/sciadv.adn6426 (PMC11186508; doi:10.1126/sciadv.adn6426)
Supplement: Supplementary file 1 — Supplementary Text Figs. S1 to S19 Legends for movies S1 to S8 [file sciadv.adn6426_sm.pdf]

Science Advances
AAAS

Supplementary Materials for

Reversible phase transformations between Pb nanocrystals and a viscous liquid-like phase

Wenjing Zheng *et al.*

Corresponding author: Haimei Zheng, hmzheng@lbl.gov; Kaiyang Niu, kniu@lbl.gov

Sci. Adv. **10**, eadn6426 (2024)
DOI: 10.1126/sciadv.adn6426

The PDF file includes:

Supplementary Text
Figs. S1 to S19
Legends for movies S1 to S8

Other Supplementary Material for this manuscript includes the following:

Movies S1 to S8

Supplementary Text

The calculation of the mechanism of the reactions:

(i) $Pb(acac)_2+2CH_3O \rightarrow Pb+2(CH_3O)(acac)$
(ii) $Pb+2CH_3O \rightarrow Pb(CH_3O)_2$
(iii) $Pb(CH_3O)_2 \rightarrow Pb+(CH_3O)_2$
(iv) $Pb(acac)_2+C_2H_4O \rightarrow Pb+(acac)_2C_2H_4O$
(v) $Pb+C_2H_4O \rightarrow Pb(C_2H_4O)$

Under the electron beam, the TEG molecules break into small fragments, such as CH_3O, C_2H_4O. We have evaluated the possible reactions (i-v) involving these small fragments. Our DFT calculations of the reaction energies are -2.00 eV/Pb for (i), -1.54 eV/Pb for (ii), -2.85 eV/Pb for (iii), 1.41 eV/Pb for (iv), and 1.84 eV/Pb for (v).

The above calculations are performed using isolated molecules in a vacuum. However, in the experiment the reactions occur under a solvation environment. To further verify the calculations, we also calculated the reaction energy using an implicit solvation model that describes the effect of electrostatics, cavitation, and dispersion on the interaction between a solute and solvent. The dielectric constant of 23.0 for TEG solvent is used. The results are as follows:

$Pb(acac)_2+2CH_3O \rightarrow Pb+2(CH_3O)(acac)$.
Vacuum: -2.00 eV/Pb   Solvation model: -2.25 eV/Pb

$Pb+2CH_3O \rightarrow Pb(CH_3O)_2$.
Vacuum: -1.54 eV/Pb   Solvation model: -1.44 eV/Pb

$Pb(CH_3O)_2 \rightarrow Pb+(CH_3O)_2$.
Vacuum: -2.85 eV/Pb   Solvation model: -2.83 eV/Pb

$Pb(acac)_2+C_2H_4O \rightarrow Pb+(acac)_2(C_2H_4O)$.
Vacuum: 1.41 eV/Pb   Solvation model: 1.28 eV/Pb

$Pb+C_2H_4O \rightarrow Pb(C_2H_4O)$.
Vacuum: 1.84 eV/Pb   Solvation model: 1.93 eV/Pb

For the system studied here, the solvation effect is small, and the conclusion obtained from vacuum calculation is not affected.

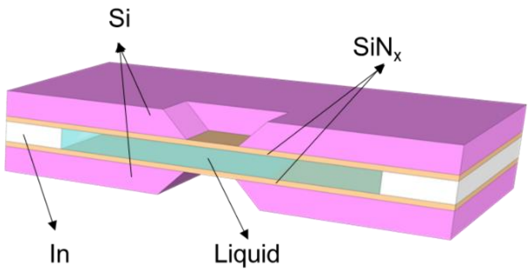

Fig. S1. Schematic illustration cut-away view of a regular liquid cell for transmission electron microscope (TEM).

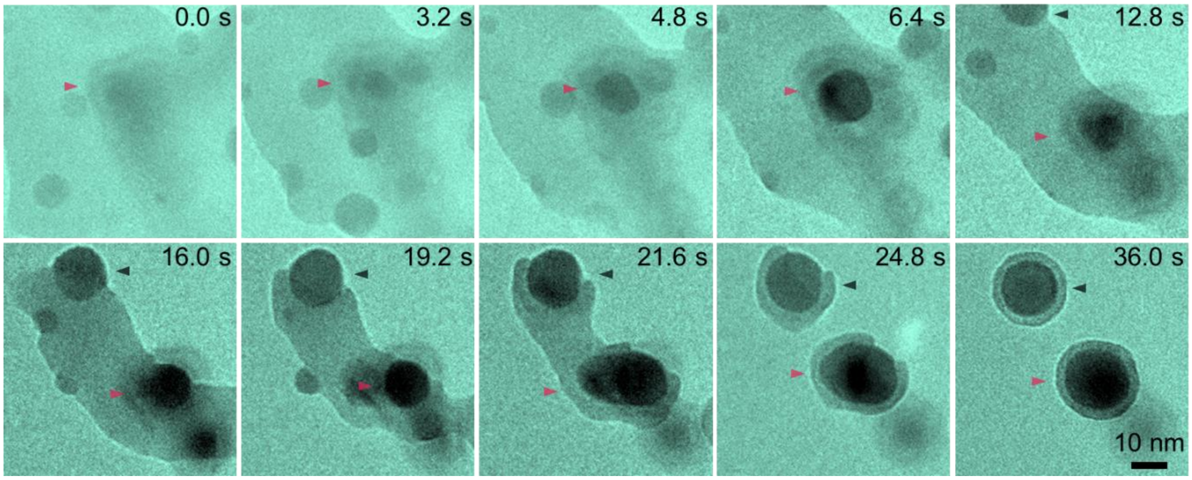

Fig. S2. In-situ synthesis of Pb nanocrystals in the liquid cell TEM. TEM image series of the formation of Pb nanocrystals (movie S1). The black and pink arrows mark two particles.

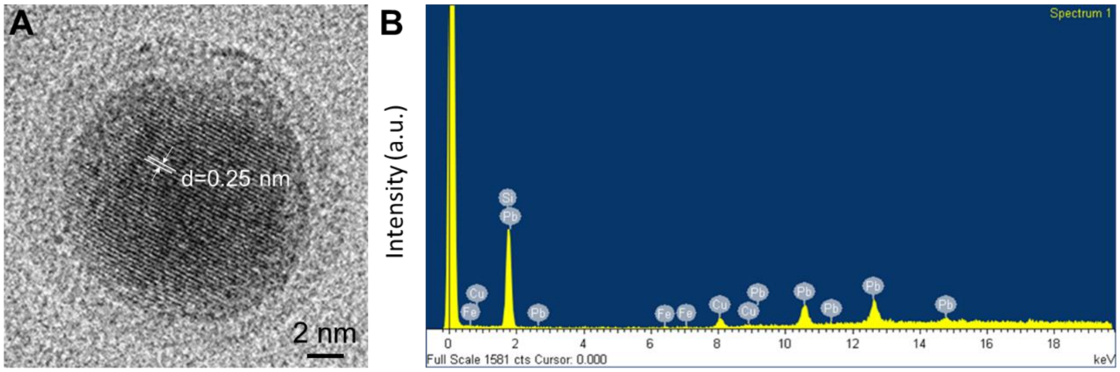

Fig. S3. Characterizations of lead core-shell nanoparticles. (A) HRTEM, the facet of the lead nanoparticle is (111). (B) EDS spectrum of the particles. The Si peak should come from the liquid cell.

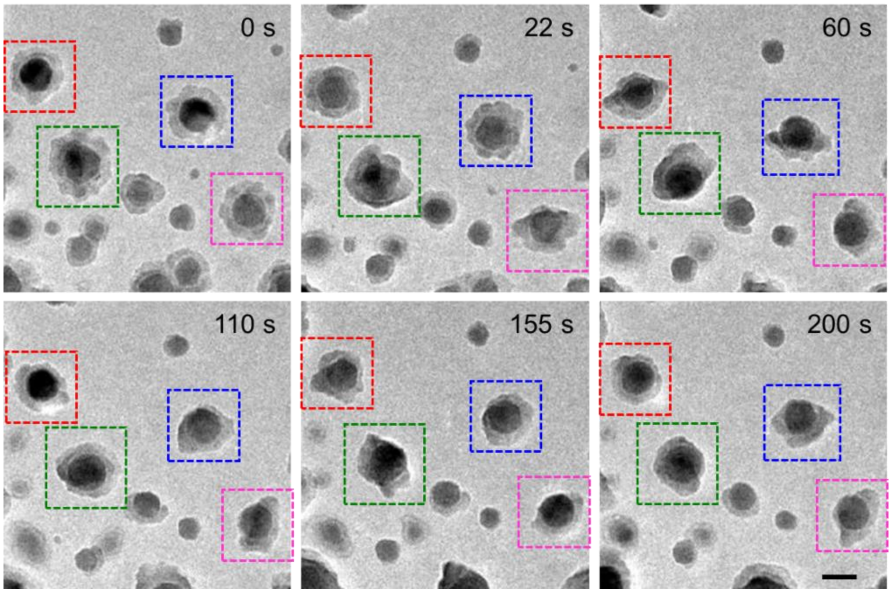

Fig. S4. Sequential TEM images showing the rotation of Pb core-shell nanoparticles. Different colored boxes highlight 4 different nanoparticles. It shows that the shell is liquid-like with changes in configurations (movie S2). The core of each nanoparticle maintains the same size, with the contrast changes indicating the nanoparticle rotation. The scale bar is 20 nm.

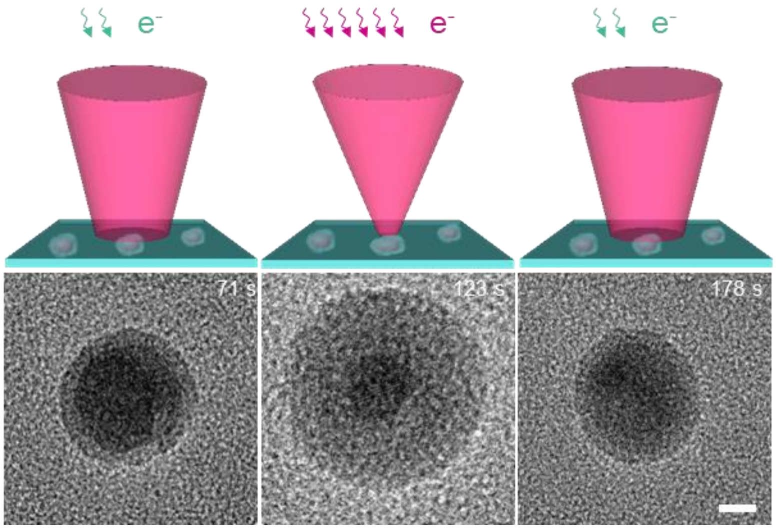

Fig. S5. Stimulate the Pb nanocrystal using a converged electron beam (movie S3). The scale bar is 2 nm.

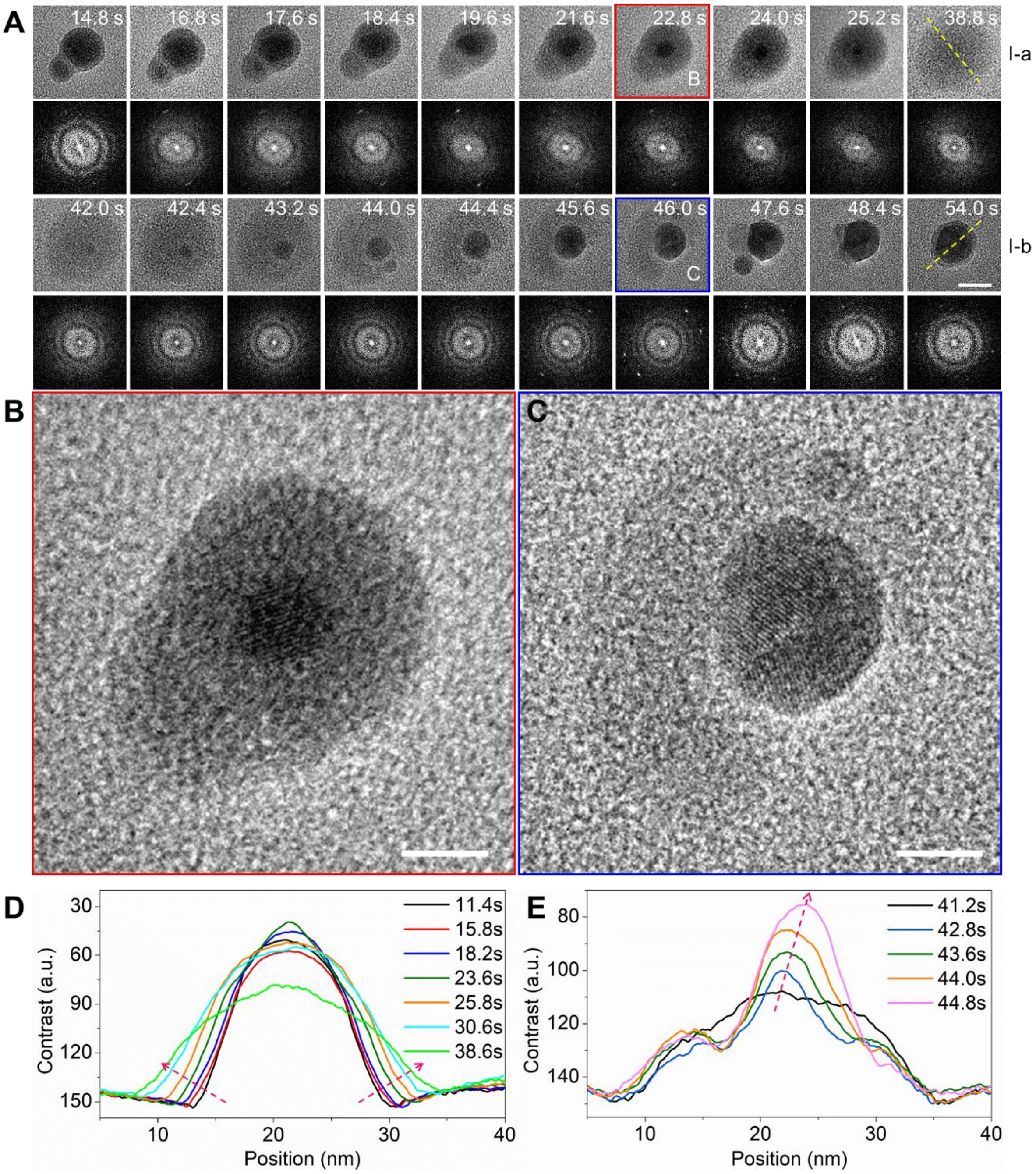

Fig. S6. The reversible transformation of Pb nanoparticles. (**A**) Sequential TEM images and the corresponding FFT for the first cycle, which corresponds to the experiment in Figure 2A and movies S3.The scale bar is 10 nm. (**B**) HRTEM of a core-shell Pb nanoparticle during the reversible transformation process at 23.0 s, which is marked as a red box in Figure S6A.The scale bar is 5 nm. (**C**) HRTEM of the core-shell Pb nanoparticle during the reversible transformation process at 45.8 s, which is marked as a blue box in Figure S6A. The scale bar is 5 nm. (**D**) The intensity of the contrast distribution of the TEM sequential images for I-a, the red dash line shows the position measured in Figure S6A 1-a. (**E**) The intensity of the contrast distribution of the TEM sequential images for I-b, the red dash line shows the position measured in Figure S6A 1-b.

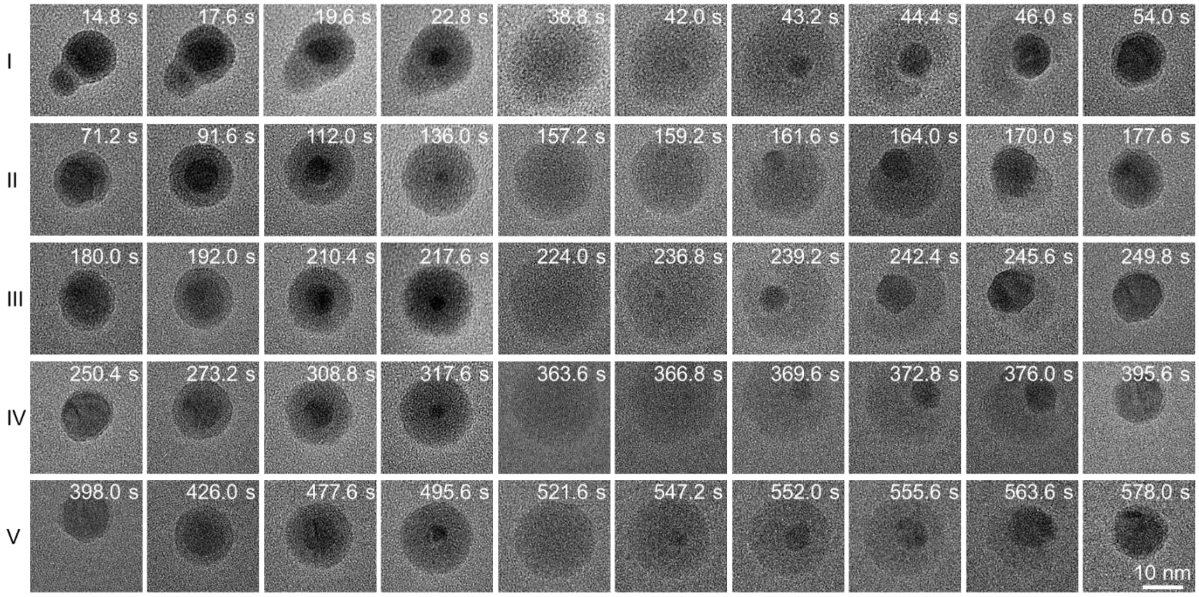

Fig. S7. The reversible transformation of Pb nanoparticles. Sequential TEM images of the reversible transformation of Pb nanoparticles. The Pb nanoparticles transformed to viscous-liquid-like phases under 3000 electrons/Å^2/s. The viscous-liquid-like phases transformed to Pb nanoparticles under 1000 electrons/Å^2/s. The five cycles are corresponding to the experiment in Figure 2A and movie S3.

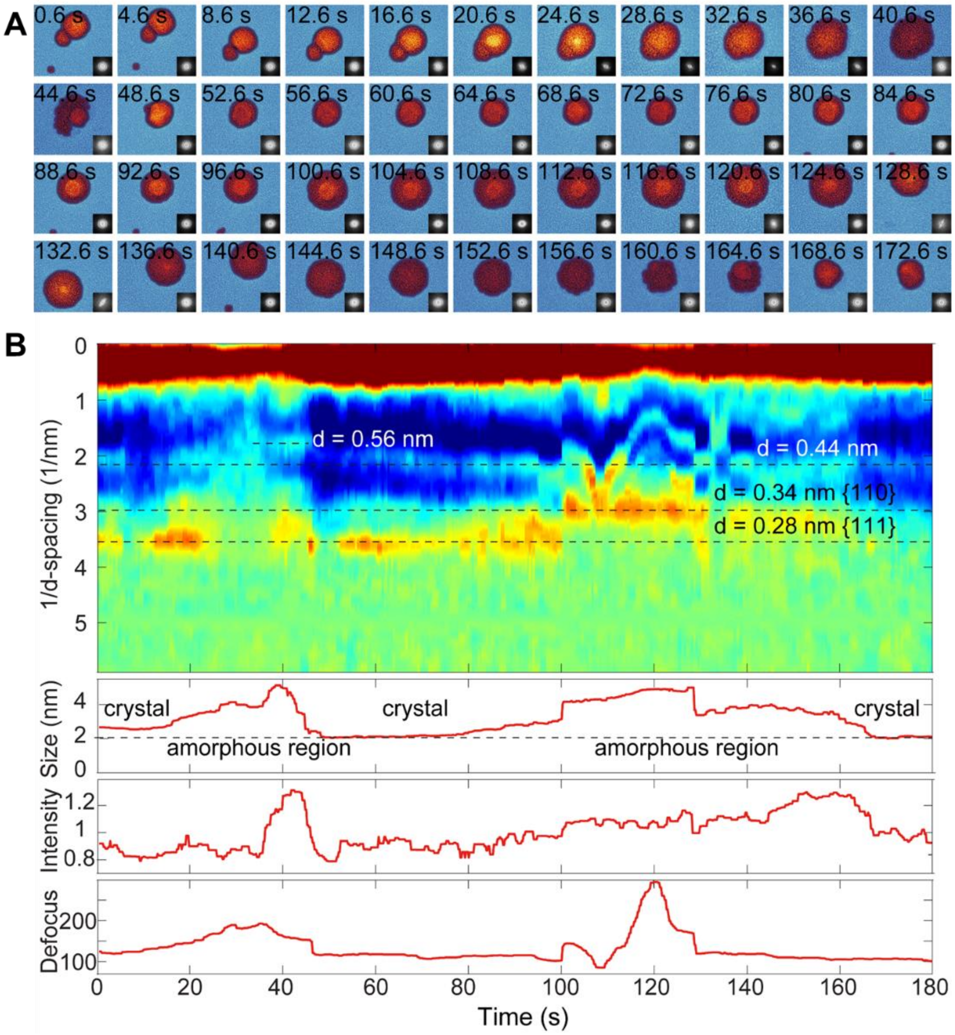

Fig. S8. Structure determination of Pb nanoparticles with the crystalline solid and viscous-liquid-like phases. (A) Sequential TEM images of the reversible transformation of Pb nanoparticles. (**B**)Determination of the lattice spacing of the Pb particle over the reversible transformations. The particle size was estimated by fitting the intensity histogram of each image (in real space) and determining the mean value and standard deviation of the background liquid signal. Intensity values lower than ~3 standard deviations of the mean background intensity were classified as belonging to the particle(s), and the total area of the particle region as a function of time is reported in Figure 4D and Figure S8B.

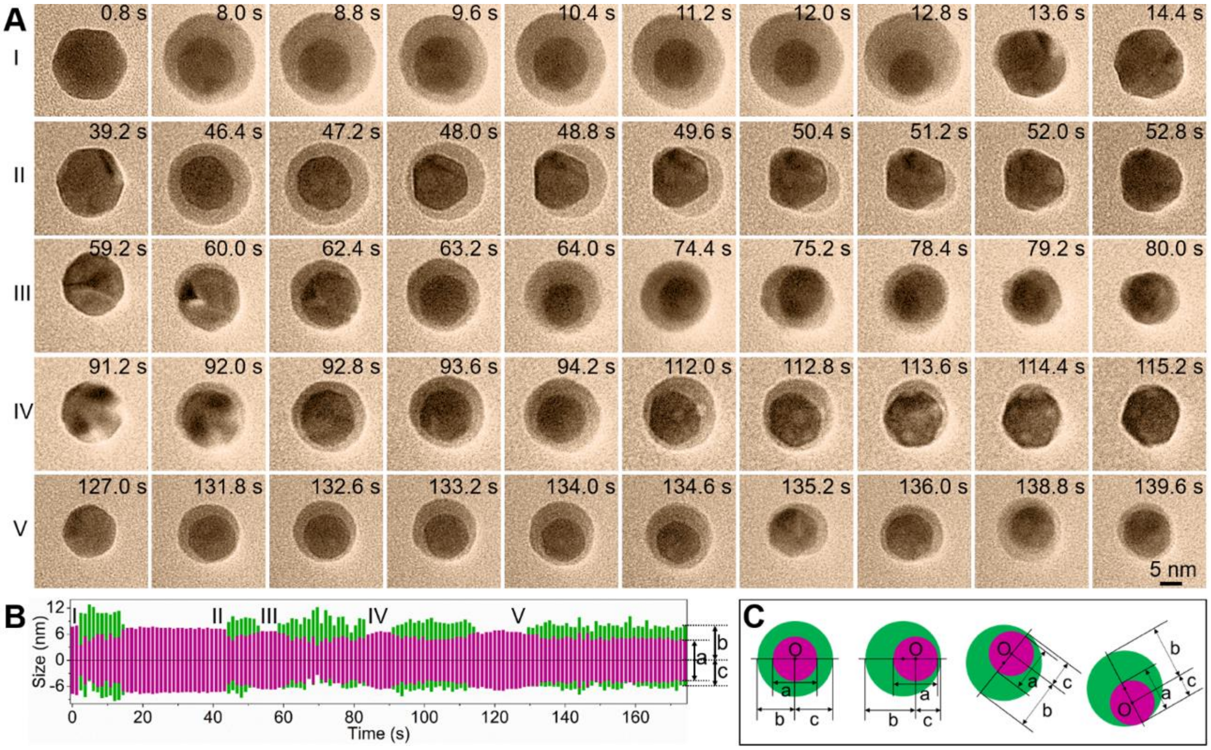

Fig. S9. Reversible Pb nanocrystal transformations under random electron beam manipulations. (**A**) Sequential TEM images of lead nanoparticles with reaction time for different cycles. (**B**) The particle sizes of the core (magenta) and shell (green) of lead nanoparticles, were measured from movie S4. (**C**) The measurement methods for Figure 4B and Figure S9B (movie S4).

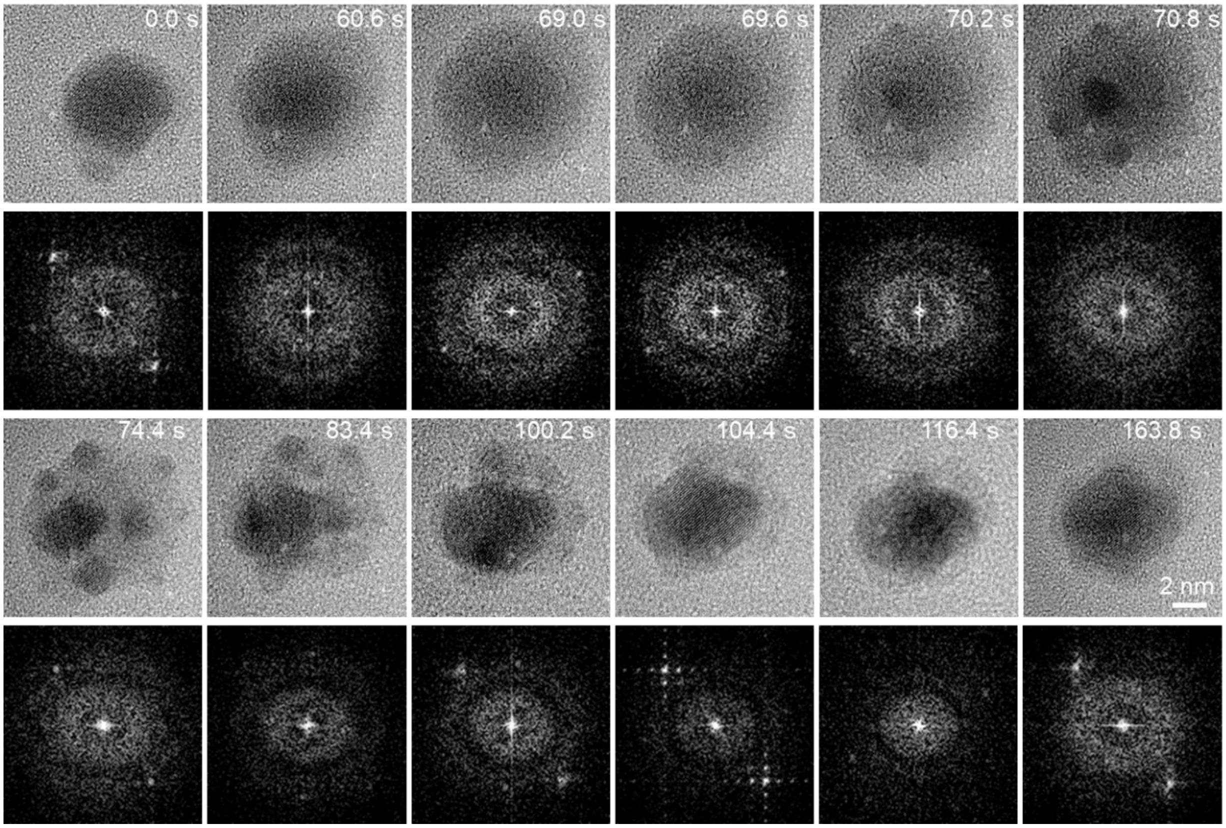

Fig. S10. Sequential TEM images and their corresponding FFT images of Pb nanoparticle transformation from crystal to jelly phase under 3500 electrons/Å^2/s, and then from jelly phase to nanoparticles under 2000 electrons/Å^2/s. (movie S5**)** Compared with the nucleation and growth under 1000 electrons/Å^2/s, the nucleation and growth under 2000 electrons/Å^2/s are quite different. There are many nuclei and the growth rate is much slower.

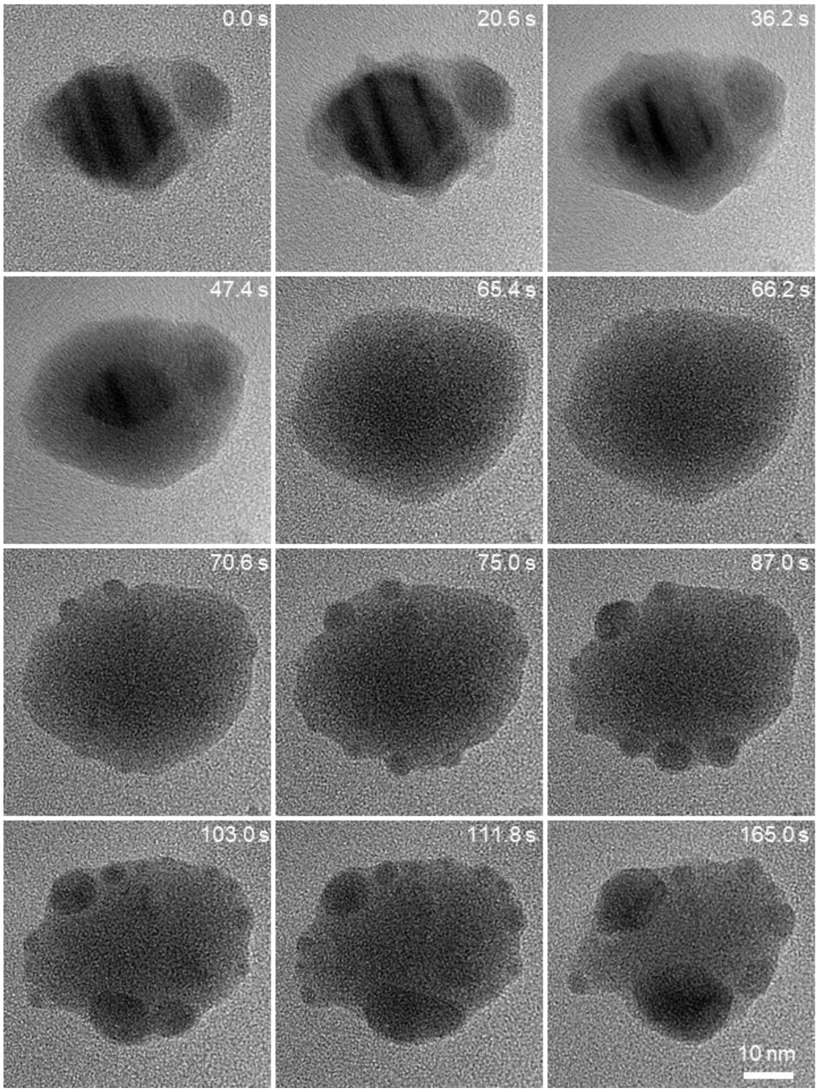

Fig. S11. Sequential TEM images of Pb nanoparticle transformation from crystal to jelly phase under 3500 electrons/Å^2/s, and then from jelly phase to nanoparticles under 2000 electrons/Å^2/s (movie S6).

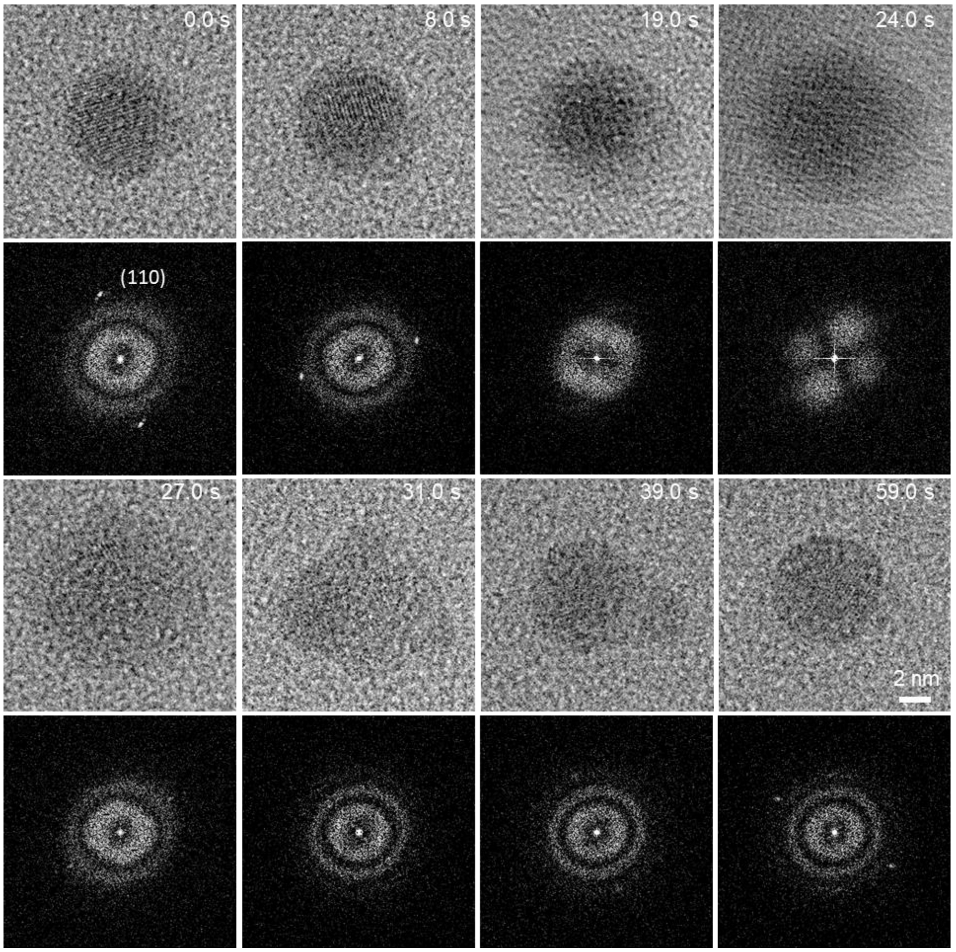

Fig. S12. Sequential TEM images and their corresponding FFT images of the reversible transformation of Pb core-shell nanoparticles with a smaller diameter, 5 nm. (movie S7)

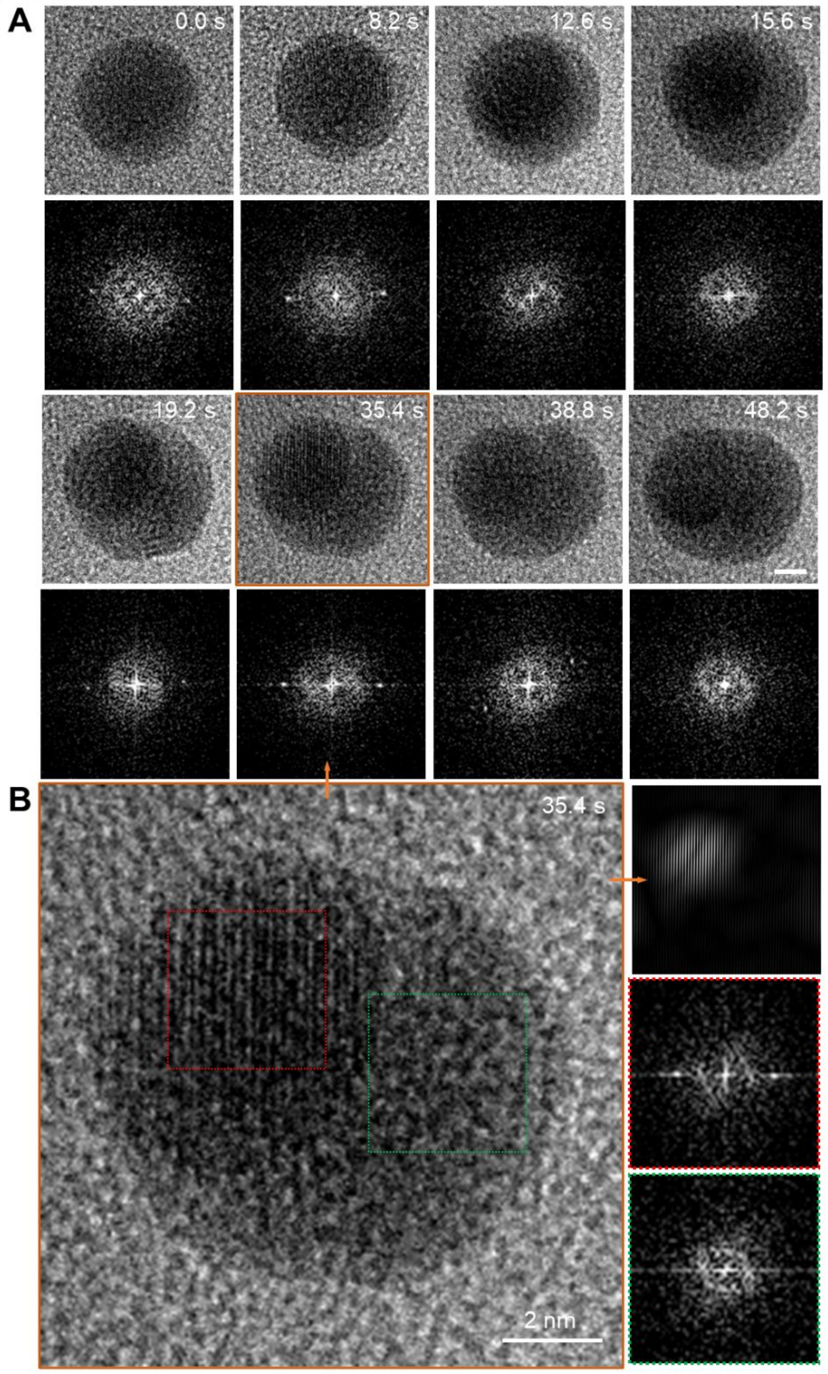

Fig. S13. The transformation of Pb nanoparticles with a diameter of 5 nm from nanoparticles. (**A**)Sequential TEM images and their corresponding FFT images. The scale bar is 2 nm. (**B**) The enlarging of the core-shell nanoparticles at 35.4 s and its reverse FFT image, corresponding FFT images of the crystal (red box) and amorphous (green box).

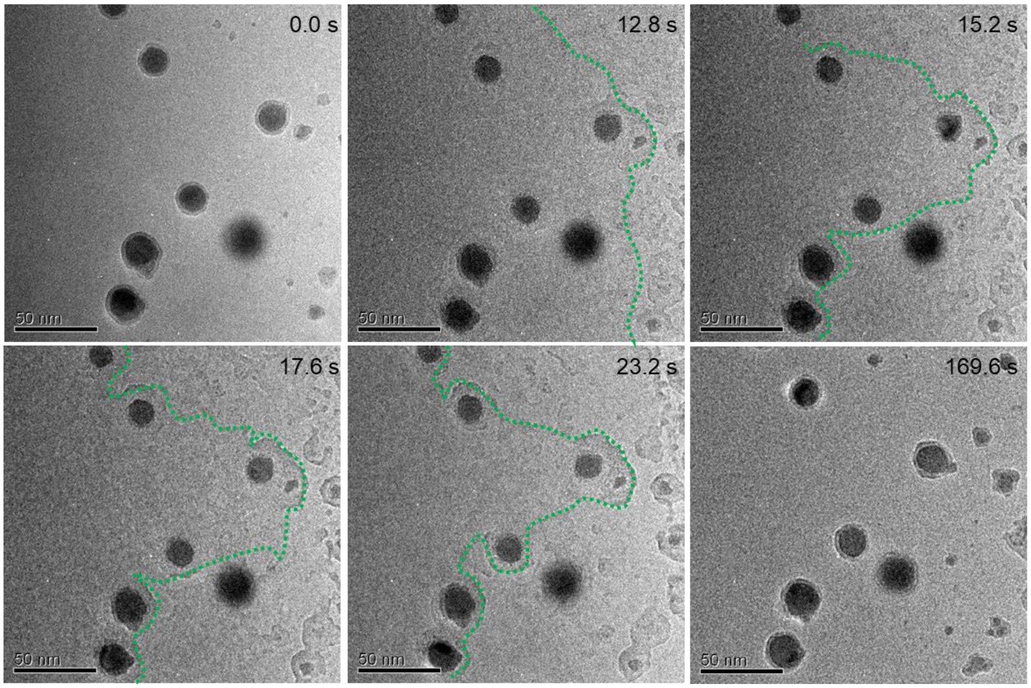

Fig. S14. Sequential TEM images with highlighted liquid edge in the cell during the transformation. (movie S8) The liquid edge was marked by a green dashed line.

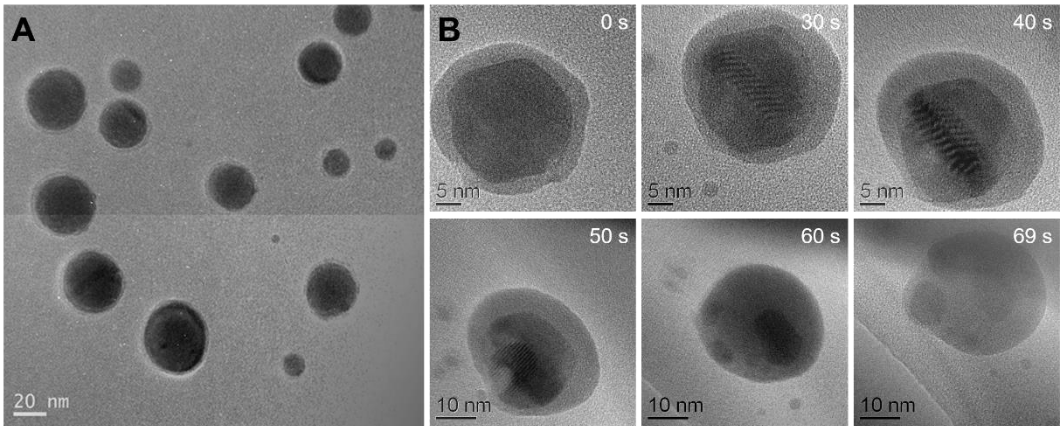

Fig. S15. TEM images of bismuth core-shell nanoparticles. (**A**) Low magnification TEM image. (**B**) Sequential images of the Bi core-shell nanoparticle transformation from crystal to jelly phase.

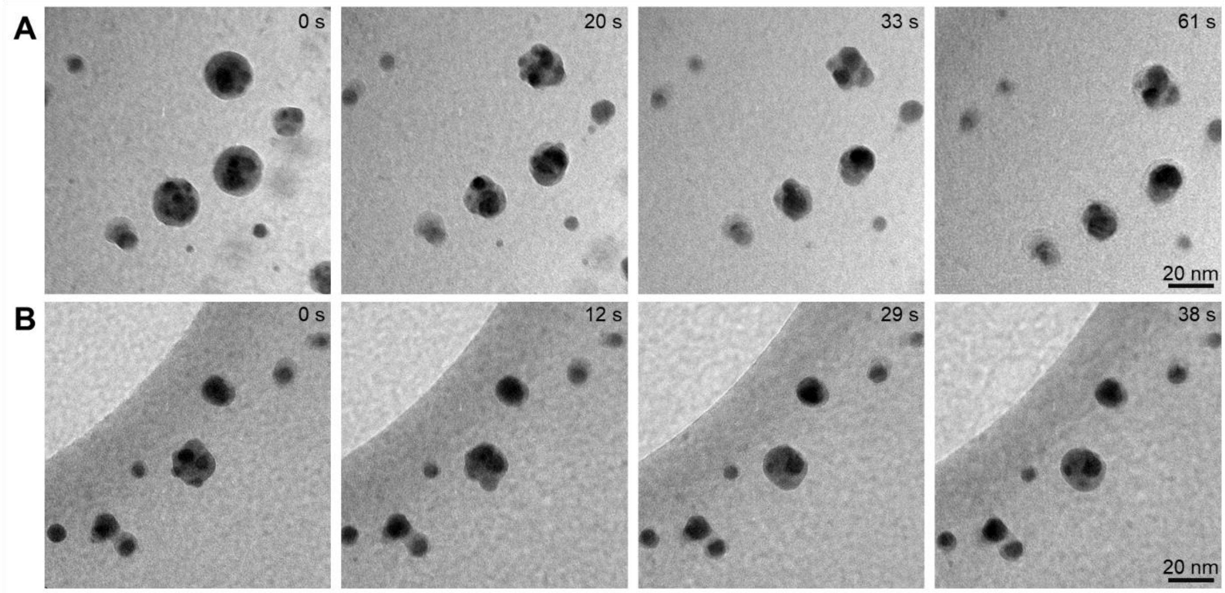

Fig. S16. The sequential TEM images of Bismuth core-shell nanoparticle transformation for different particles in selected area (A) and selected area (B).

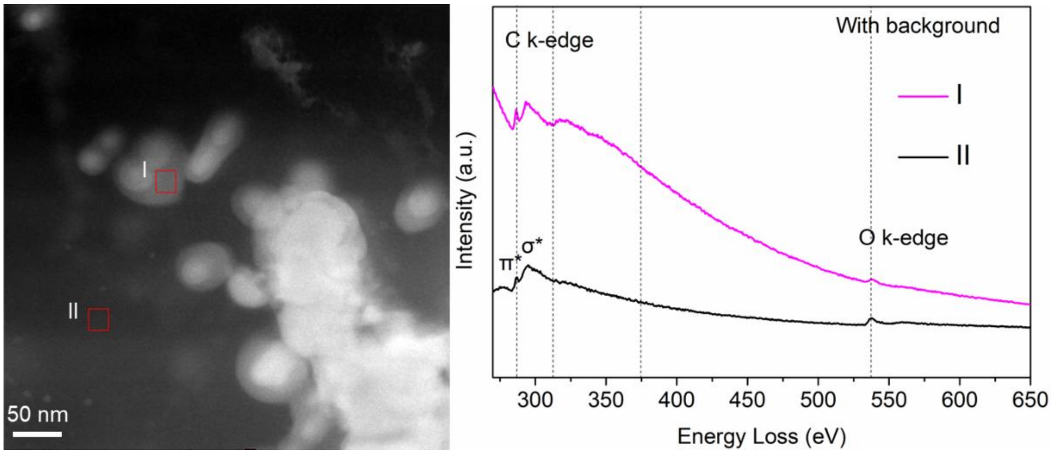

Fig. S17. High-angle annular dark-field scanning transmission electron microscope (HAADF-STEM) image in liquid cell and corresponding carbon K-edge EELS marked on STEM image. The amorphous (I) area of the Pb nanoparticle and the liquid (II) area. There is a wide peak in the I curve, which may be caused by the fragments of TEG. Oxygen is also found in the shells of nanoparticles.

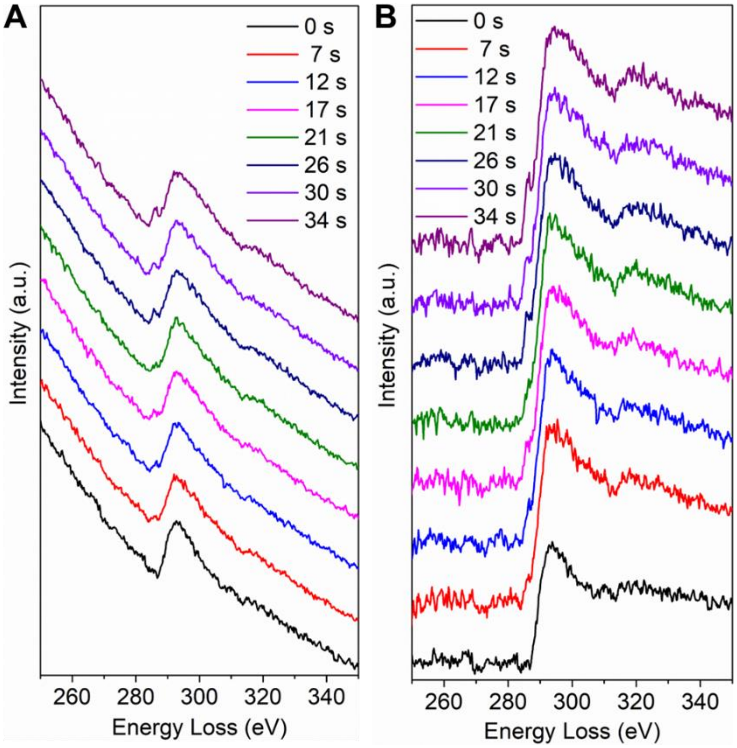

Fig. S18. The electron energy loss spectroscopy (EELS) spectra of carbon K-edge for triethylene glycol (TEG) under beam with time. (**A**) With background. (**B**) After background removal. The intensity of the wide peak around 320 eV increases with time, which may result from the increased TEG fragments (CHO_3, C_2H_4O, etc.).

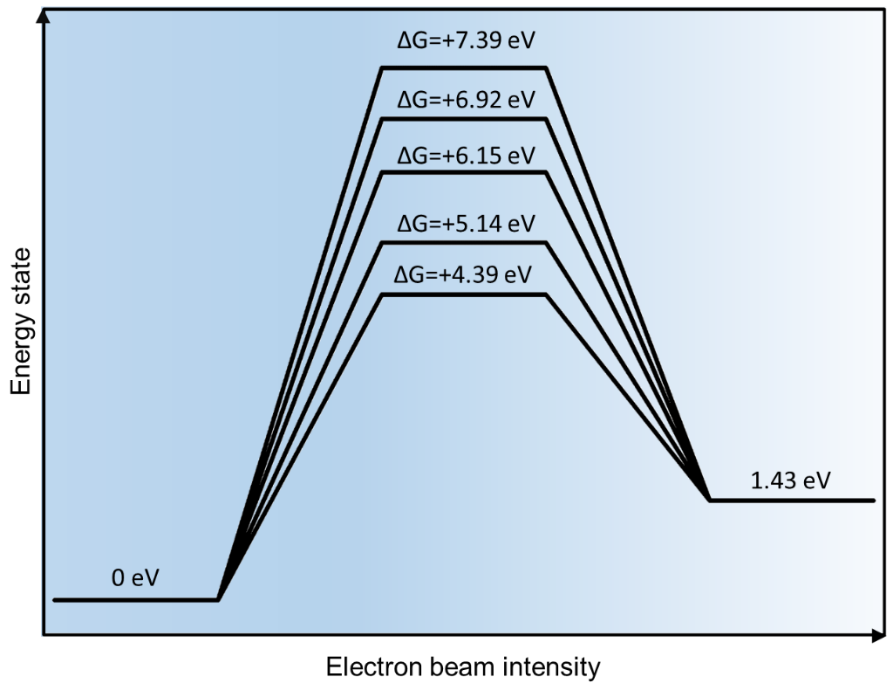

Fig. S19. The calculation of the energy level of the Pb particles in different states. The energy of an isolated $Pb(CH_3O)_2$ is set to zero. The energy states are the distance of the Pb crystal with 120%, 140%, 160%, 180%, and 200%.

Movie S1.

Lead core-shell nanocrystals generation.

Movie S2.

Rotation of Pb core-shell nanoparticles.

Movie S3.

Reversible transformation of lead core-shell nanocrystals between a solid phase and a viscous-liquid-like phase under high-dose-rate electron beam (3000 electrons/$Å^2$/s) or low-dose electron beam (1000 electrons/$Å^2$/s), respectively.

Movie S4.

Reversible transformation of lead core-shell nanocrystals between a solid phase and a viscous-liquid-like phase under random electron beam manipulations (high-dose-rate electron beam, 3000 electrons/$Å^2$/s, or low-dose electron beam, 1000 electrons/$Å^2$/s), respectively.

Movie S5.

Reversible transformation of lead core-shell nanocrystals between a solid phase and a viscous-liquid-like phase under high-dose-rate electron beam (3500 electrons/$Å^2$/s) or low-dose electron beam (2000 electrons/$Å^2$/s), respectively.

Movie S6.

Reversible transformation of lead core-shell nanocrystals between a solid phase and a viscous-liquid-like phase under high-dose-rate electron beam (3500 electrons/$Å^2$/s) or low-dose electron beam (2000 electrons/$Å^2$/s), respectively.

Movie S7.

Reversible transformation of lead core-shell nanocrystals with a smaller diameter, 5 nm, between a viscous-liquid-like phase and a jelly phase under a high-dose-rate electron beam (3000 electrons/$Å^2$/s) or low-dose electron beam (1000 electrons/$Å^2$/s), respectively.

Movie S8.

Reversible transformation of multiple lead core-shell nanocrystals between a solid phase and a viscous-liquid-like phase under random electron beam manipulations (high-dose-rate electron beam, 3000 electrons/$Å^2$/s, or low-dose electron beam, 1000 electrons/$Å^2$/s), respectively.
